# Fragment-based computational design of antibodies targeting structured epitopes

**DOI:** 10.1126/sciadv.abp9540

**Published:** 2022-11-11

**Authors:** Mauricio Aguilar Rangel, Alice Bedwell, Elisa Costanzi, Ross J. Taylor, Rosaria Russo, Gonçalo J. L. Bernardes, Stefano Ricagno, Judith Frydman, Michele Vendruscolo, Pietro Sormanni

**Affiliations:** ^1^ Centre for Misfolding Diseases, Yusuf Hamied Department of Chemistry, University of Cambridge, Cambridge CB2 1EW, UK.; ^2^ Department of Biology, Stanford University, Stanford, CA, USA.; ^3^ Department of Bioscience, Università degli Studi di Milano, Milano 20133, Italy.; ^4^ Department of Pathophysiology and Transplantation, Università degli Studi di Milano, Milano 20122, Italy.; ^5^ Institute of Molecular and Translational Cardiology, IRCCS Policlinico San Donato, Milan 20097, Italy.

## Abstract

De novo design methods hold the promise of reducing the time and cost of antibody discovery while enabling the facile and precise targeting of predetermined epitopes. Here, we describe a fragment-based method for the combinatorial design of antibody binding loops and their grafting onto antibody scaffolds. We designed and tested six single-domain antibodies targeting different epitopes on three antigens, including the receptor-binding domain of the SARS-CoV-2 spike protein. Biophysical characterization showed that all designs are stable and bind their intended targets with affinities in the nanomolar range without in vitro affinity maturation. We further discuss how a high-resolution input antigen structure is not required, as similar predictions are obtained when the input is a crystal structure or a computer-generated model. This computational procedure, which readily runs on a laptop, provides a starting point for the rapid generation of lead antibodies binding to preselected epitopes.

## Teaser

A combinatorial method can rapidly design nanobodies that bind with nanomolar affinity predetermined epitopes.

## INTRODUCTION

Antibodies are key tools in biomedical research and are increasingly used to diagnose and treat a wide range of human diseases. Now, there are over 120 antibodies approved or undergoing regulatory review in the United States and Europe (*1*). Existing antibody discovery methods have been widely successful, but still have important limitations (*2*). Extensive laboratory screenings are required to isolate those antibodies binding to the intended target, which can be time consuming and costly. Some classes of hard targets remain, including some receptors and channels, proteins within highly homologous families, aggregation-prone peptides, and disease-related short-lived protein aggregates (*3*, *4*). Most notably, it is often highly challenging to obtain antibodies targeting preselected epitopes. Screening procedures typically select for the tightest binders, which usually occur for immunodominant epitopes, thus disfavoring the discovery of antibodies with lower affinities but binding to functionally relevant sites (*5*). Furthermore, screening campaigns often yield antibodies with favorable binding affinity but otherwise poor biophysical properties, such as stability, solubility, and production yield, which may hinder their development into effective reagents. Computational antibody design has the potential to overcome these limitations by markedly reducing time and costs of antibody discovery and, in principle, allowing for a highly controlled parallel screening of multiple biophysical properties. Moreover, rational design inherently enables the targeting of specific epitopes.

Most available methods for the design of binding proteins rely at least in part on the minimization of a calculated interaction free energy, through the sampling of the mutational space and the conformational space (*2*, *6*, *7*). The nature of these calculations, which are based on molecular modeling and classical force fields, and the challenges of achieving exhaustive sampling make simulations rather imprecise and highly resource intensive. For these reasons, the de novo design of antibody binding has generally met low success rates and required recursive experimental screenings and large libraries (*5*, *8*–*10*). Computational design of binding has been most successful in synergy with in vitro affinity maturation and, in particular, when applied to miniproteins (*11*–*13*). The small size of these miniproteins is amenable to the high-throughput gene synthesis required to experimentally screen designed candidates on a massive scale, and their rigidity reduces the need for accurate conformational sampling. However, antibody domains are considerably larger and bind their target using complementarity-determining regions (CDRs) located within hypervariable loops on the antibody surface, which are often extended and highly flexible.

Here, we describe a novel method to design antibody CDR loops targeting epitopes for which a structure is available, from either an experimentally determined structure or a computational model. Designed CDRs are then grafted onto antibody scaffolds and further optimized computationally for solubility and conformational stability. Novel antibody-antigen interactions are designed by combining together protein fragments identified as interacting with each other within known protein structures.

## RESULTS

### De novo CDR design strategy

To overcome some of the limitations of molecular modeling mentioned above, in particular those associated with the approximations of the interatomic interactions, we exploited the availability of large structural databases to implement a fragment-based procedure to design CDRs (paratope) complementary to a target epitope. To implement this idea, we compiled from the nonredundant Protein Data Bank (PDB) a database of CDR-like fragments and corresponding antigen-like regions, which we call AbAg database. CDR-like fragments are defined as linear motifs structurally compatible with an antibody CDR loop, which may be found in any protein structure in the PDB. Conversely, antigen-like regions comprise those residues interacting with CDR-like fragments in the context of the protein where the CDR-like fragment is originally found (see Materials and Methods).

**Fig. 1.**
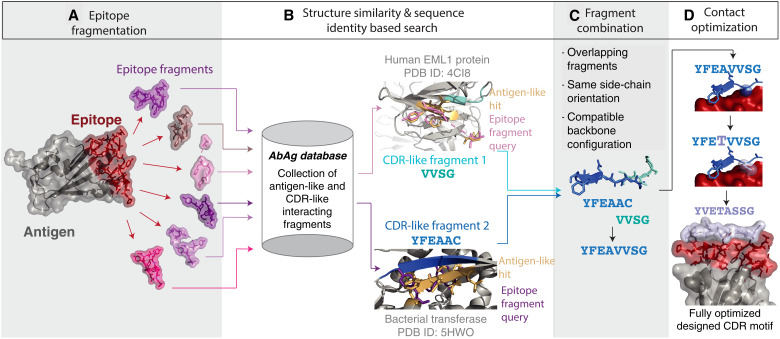
Workflow for the combinatorial structure-based CDR design strategy. (**A**) The antigen structure is shown in gray, with the epitope of interest highlighted in red. At this step, the epitope is fragmented into its structural fragments, both in a linear mode and in a surface-patch mode that yields also noncontiguous fragments (see text). Some example fragments are pointed by red arrows. (**B**) These fragments are used as queries for a structural search against a custom database of structures of antigen-like and CDR-like interacting fragments. Hits are selected on the basis of structural and sequence similarity with the query epitope fragments, and two example hits are depicted: an epitope fragment (pink top example, purple lower example) matching antigen-like fragments (yellow) interacting with a CDR-like fragment (cyan top example, blue lower example). These two examples originate respectively from the structures of human EML1 protein and of a bacterial transferase, as antigen-like and CDR-like fragments may be found in any structure from the PDB. (**C**) When possible, identified CDR-like fragments are joined together. Here, the overlapping CDR-like fragments from B are merged as they meet the stated compatibility criteria. (**D**) The sequence of the designed CDR fragment resulting from the merging is optimized to increase the probability of favorable CDR-epitope contacts. The final fully optimized designed CDR motif can then be grafted onto suitable antibody frameworks. The example in this figure corresponds to the designed binding motif within the CDR3 of DesAb-RBD-C1 targeting the ACE2-binding site on the RBD domain.

Given the structure of a target epitope, the database can be searched to identify antigen-like regions similar to this epitope or to fragments of it. In this way, the CDR-like fragments interacting with the identified antigen-like regions may also interact with the target epitope. To perform this search, the structure of the input epitope is fragmented using two different strategies (Fig. 1A): (i) a linear fragmentation, which generates fragments of at least four consecutive residues, and (ii) a surface-patch fragmentation, which takes each residue and yields the closest *n* ≥ 4 solvent-exposed residues in the three-dimensional structure of the epitope. The reason for this choice is that *n* < 4 results in a substantially slower search, and small fragments are unlikely to capture enough of the epitope complexity to yield CDR-like fragments that would actually bind to the target epitope. These two approaches allow for covering a wider search space, as the first one conducts an exhaustive search for contiguous epitopes, whereas the second one is more suitable for conformational epitopes comprising multiple segments, which are generally distant in the sequence space. Next, each epitope fragment is compared to the antigen-like regions to identify those with compatible backbone structure and similar sequence. More specifically, the search is carried out with the Master algorithm (*14*), and the comparison is based on the root mean square deviation (RMSD) of the full backbone and on sequence similarity (see Materials and Methods). Therefore, a hit antigen-like region is similar to its query epitope fragment in both sequence and structure. In practice, the fragmentation is carried out starting from large fragments (i.e., from the full region defined as epitope) and moving to smaller ones for a minimum size of four residues. Most commonly, no hits are found for larger fragments, while many hits are typically found for smaller ones (*n ≤ 6*).

Because of the nature of the AbAg database, this procedure yields those CDR-like fragments that interact with the identified antigen-like fragments (Fig. 1B). These CDR-like structures are then rotated to match the orientation of the epitope, by superimposing each antigen-like region, together with its interacting CDR-like fragments, to the matching part of the epitope (Fig. 1 and fig. S1). When possible, different CDR-like fragments whose backbones are partly overlapping and compatible with a single longer CDR loop are joined together to yield longer interacting motifs (Fig. 1C; see Materials and Methods).

Some of the original interactions of each CDR-like fragment may be affected when this fragment is transferred onto the epitope, for instance, if the sequence of the antigen-like region is not identical to the corresponding epitope sequence, or if the epitope side chains are found in different conformations (Fig. 1D). Similarly, new interactions may arise when a CDR-like fragment forms contacts with parts of the epitope that were not matched onto its antigen-like region. To overcome potential issues arising from these suboptimal interactions, we implemented a side-chain optimization procedure that seeks to maximize the number of favorable interactions between the CDR-like fragment and the antigen. Briefly, for each CDR-like side chain with interactions different, or additional, to those found in the original hit, a structural neighborhood is defined by taking the backbone coordinates of all contacting residues (see Materials and Methods). These residues are then used as a query to interrogate the AbAg database, retrieving as hits those CDR-like side chains that better match the native local environment of the epitope, therefore increasing the total number of favorable interactions to yield a fully optimized designed CDR motif (Fig. 1D; see Materials and Methods).

Typically, multiple CDR motifs are designed in this way for a given input epitope, as multiple CDR-like fragments are usually identified as suitable starting points for the combination and the optimization procedures. Therefore, all possible CDR motif candidates generated for the input epitope are ranked according to the total number of favorable interactions, the number of interactions that could not be optimized, and a solubility score calculated with the CamSol method (*15*).

**Fig. 2.**
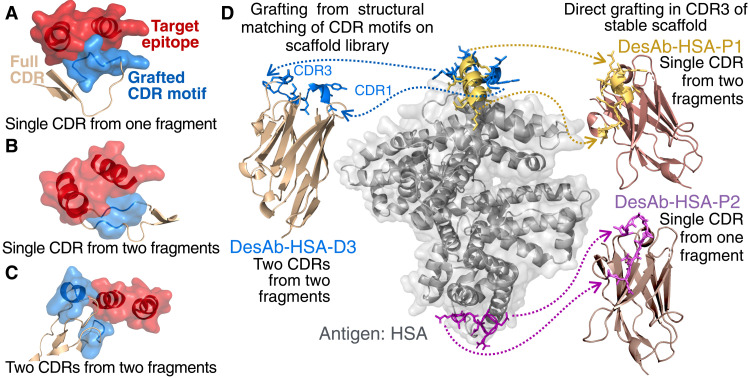
Grafting of designed CDR motifs onto antibody scaffolds. (**A** to **C**) Three examples of how designed CDR motifs can be grafted in different ways. The epitope is shown in red, and the designed CDR motifs are shown in light blue. These are grafted onto structurally matched CDR loops (light brown). In (A), a single motif is grafted in a loop; in (B), two motifs are grafted on the same loop; and in (C), two motifs are grafted in two different loops from the same Fv domain. Multiple CDR-like fragments are joined in a single motif when overlapping (like in Fig. 1C) or, if not overlapping, may still be grafted in the same CDR loop as shown in (B). (**D**) The structure of HSA is shown in gray, and the designed CDR motifs selected for experimental validation are shown in blue, yellow, and purple docked onto their respective epitopes. Two fragments (blue) are grafted into separate CDRs (CDR1 and CDR3) of an antibody scaffold, which they match structurally (PDB 4DKA). The resulting design is DesAb-HSA-D3 (Table 1). The yellow and purple motifs are instead grafted into the CDR3 of a scaffold resilient to CDR3 substitutions to yield DesAb-HSA-P1 and DesAb-HSA-P2. The motif grafted onto DesAb-HSA-P1 comprises two fragments joined together as in Fig. 1C. DesAb structural models were obtained with the SAbPred webserver (*51*).

Top-ranking, designed CDR motifs can then be grafted into an antibody scaffold (Fig. 2). Our pipeline can structurally match the generated motifs either to complete CDRs or entire antibody structures (specifically Fv regions), which can result in longer CDR loops harboring multiple motifs, or in multiple motifs being grafted in different CDR loops of the same Fv region (Fig. 2, A to C; see Materials and Methods). If needed, any new interactions between the grafted antibody scaffold and the antigen are optimized using the side-chain optimization procedure described above. Furthermore, as an alternative to this structural matching, designed CDR motifs can also be grafted directly into an antibody scaffold that is already known to be highly tolerant to loop replacements. In this work, we tested experimentally both approaches (Fig. 2D).

To validate our design strategy, we tested it experimentally on single-domain antibodies, because of their monomeric nature, ease of production in prokaryotic systems, and small size (*16*). Nonetheless, the computational design pipeline described here can readily be applied to other antibody fragments, including whole Fv regions, on which designed CDR motifs can be structurally matched and grafted in the same way on either heavy- or light-chain CDRs.

### Description of designs and biophysical characterization

We designed six single-domain antibodies for three different antigens by exploring two grafting strategies: the direct grafting of the designed CDRs onto stable scaffolds and the matching of the designed CDRs to a scaffold that is structurally compatible with them. The first strategy provides the opportunity to test the de novo CDR design procedure by minimizing possible complications arising from the grafting, while the second is a more complex approach that allows to design multiple CDR loops onto a scaffold structurally matched to the epitope. Two designed single-domain antibodies (DesAbs) target the severe acute respiratory syndrome coronavirus 2 (SARS-CoV-2) spike protein receptor-binding domain (RBD), three human serum albumin (HSA), and one pancreatic bovine trypsin (Table 1). HSA and trypsin were selected for the initial validation. Both proteins are available off the shelf, and binding of therapeutic proteins to HSA is a key determinant of pharmacokinetics. Therefore, single-domain antibodies targeting HSA may provide a tool for enhancing the half-life of biologics (*17*). Conversely, trypsin offers the opportunity of testing the design strategy on poorly accessible concave epitopes harboring an active site. The RBD of SARS-CoV-2 exemplifies the power of targeting-specific epitopes, as binding to regions overlapping with, or close to the ACE2 receptor binding site, while avoiding glycosylation sites, is known to yield neutralizing antibody candidates, which would sterically hinder virus binding to the human cell receptor (*18*). In this case, we used as starting point for the design the first-released cryo–electron microscopy (cryo-EM) model of the SARS-CoV-2 spike protein in the prefusion conformation (PDB ID 6VSB) (*19*). The reason for this choice was to assess how the design strategy performs with a lower-resolution structure used as input. Specifically, we ran the design on the surface of the up RBD around the ACE2-binding region, which has some regions of low resolution (~6 to 8 Å) (*19*) and several missing residues in the model.

**Table 1.** DesAbs used in this study.

	**Target antigen**	**Designed CDR**	Target epitope*	**Scaffold (PDB)^†^**	***T*_m_ (°C)^‡^**	***K*_d_ (nM)^§^**
DesAb-HSA-P1	HSA	IQKSLQTAESIL	575–582	6Z3X	82.5	120
DesAb-HSA-P2	HSA	AQAGNAEEAE	71–80	6Z3X	80	380
DesAb-HSA-D3	HSA	ELYALI (CDR1) KFASPDGS (CDR3)	542–546, 574–580	4DKA	67.5	180
DesAb-Tryp	Trypsin	QSGYHF	698–702	6Z3X	78.5	1800
DesAb-RBD-C1	Spike RBD	GSSATEVY	449,453,492– 497,500	6Z3X	77.5	210
DesAb-RBD-C2	Spike RBD	VVADLSV	353–359	6Z3X	80	130

*Residue numbering as in PDB entries 1AO6 chain B (HSA), 1S0Q chain (trypsin), and 6VSB chain A (spike RBD).

†PDB ID of scaffold in whose loop the designed CDRs are grafted. 6Z3X is from this study.

‡Melting temperature rounded to the closest 0.5°C to reflect the accuracy of the thermal shift assay used (see fig. S3C).

§As measured with BLI, rounded to the closest 10 nM with exception of DesAb-Tryp, which was rounded to 100 nM (fig. S6)

All DesAbs expressed well in *Escherichia coli* were obtained to high purity and showed circular dichroism (CD) spectra fully compatible with a well-folded variable heavy (VH) domain (fig. S2; see Materials and Methods). All designs were highly stable, with a melting temperature at par or better than that of immune system–derived nanobodies (Table 1 and fig. S2C) (*20*).

Two of the three anti-HSA single-domain antibodies, DesAb-HSA-P1 and DesAb-HSA-P2 (Table 1 and Fig. 2D), consisted in designed CDR motifs grafted in place of the CDR3 of a previously characterized single-domain antibody scaffold highly amenable to CDR3 substitutions (*21*, *22*) (table S1). The third design, DesAb-HSA-D3, was made by structurally matching two separate CDR-like candidates onto two CDR loops of a nanobody scaffold identified as highly compatible with these two binding motifs (Fig. 2D; see Materials and Methods).

We note that this pipeline recovered and scored highly the sequences of the CDRs of an existing nanobody targeting HSA, called Nb.B201, whose structure in complex with the antigen was processed during the AbAg database construction (table S4). While this observation serves as an in silico consistency check for our design method, when selecting fragments for all designs used in this study, we excluded fragments originating from antibodies or peptides already known to bind to the target antigen, and in the case of HSA, we also selected different epitopes.

**Fig. 3.**
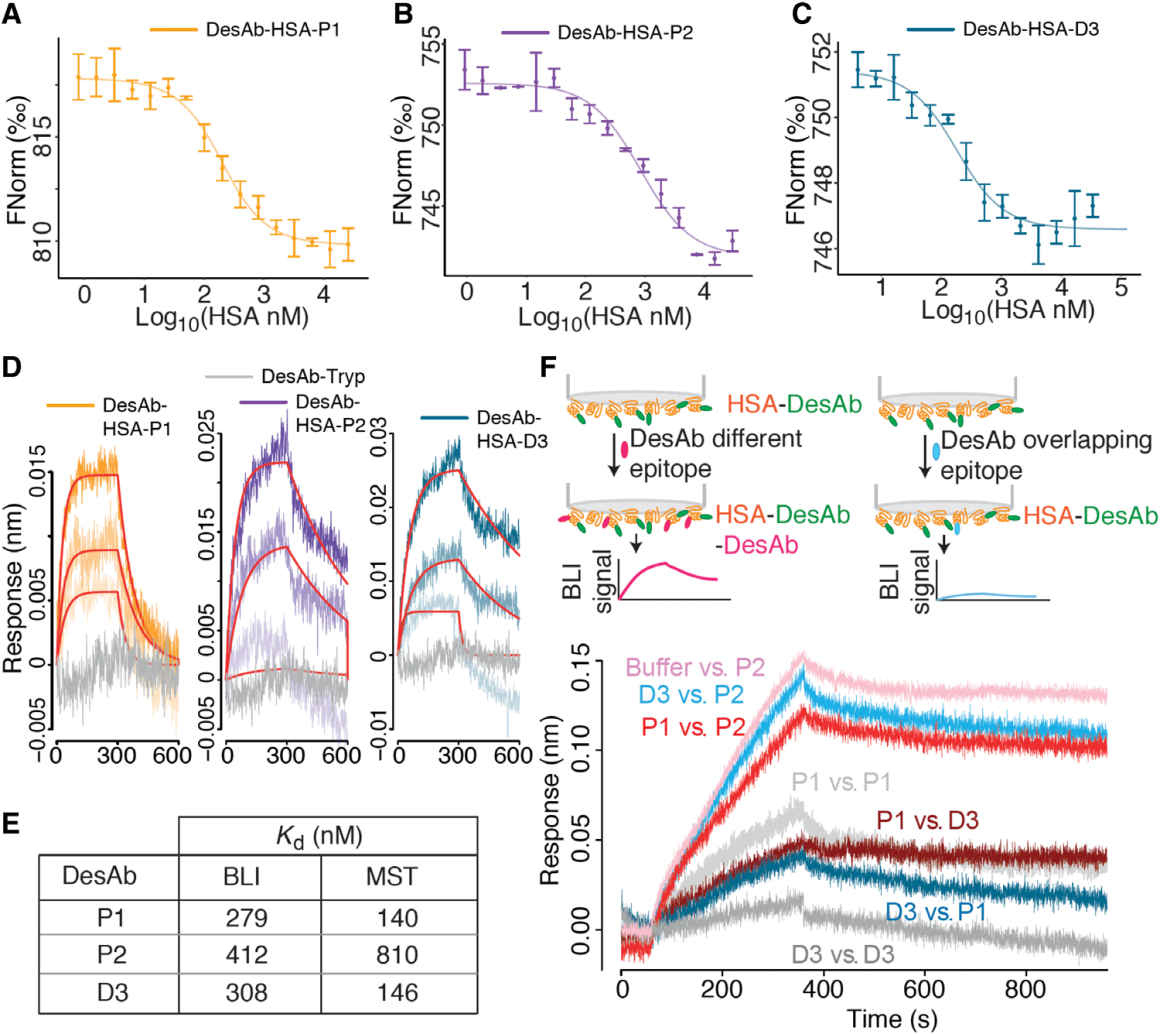
The anti-HSA DesAbs bind their target and compete for binding to overlapping epitopes. (**A** to **C**) Microscale thermophoresis (MST) of fluorescently labeled DesAbs (70 nM) in the presence of increasing concentrations of HSA (*x* axis). Data points are mean and SDs of three replicates; data were fitted with a single-site binding model. (**D**) BLI binding traces (association and dissociation) obtained with APS sensors loaded with HSA. Association was monitored in wells containing 1, 0.5, and 0.25 μM DesAbs. DesAb-Tryp (1 μM) is shown in gray and was used as control for nonspecific binding to the sensor. (**E**) Table with the dissociation constants (*K*_d_) obtained for the three DesAbs by fitting the BLI and MST data. (**F**) Binding competition experiment at the BLI. APS sensors were loaded with HSA, dipped in wells containing 5 μM of a first DesAb X1 (see Materials and Methods), and moved in buffer wells for 1 min, then into wells containing 5 μM of a second DesAb X2, and finally back to buffer wells. Curves are labeled with “X1 versus X2” to identify the anti-HSA DesAbs used. The plot shows the last three steps, and reference sensors monitoring the background dissociation of X1 during these steps were subtracted from the traces shown here. The traces P1 versus P1 and D3 versus D3 were taken as positive controls for the competition, and the small signal observed is due to the facts that not all epitopes are occupied by the first DesAb (X1) and that this is dissociating in the background. The trace Buffer versus P2 was taken as a negative control for the competition.

Binding to HSA was measured in solution with microscale thermophoresis (MST), which yielded *K*_d_ values ranging from 140 to 800 nM (Fig. 3, A to C and E), while a control single-domain antibody showed extremely weak signal in this assay (fig. S4A). To put this in context, the Nb.B201 nanobody, which was isolated with yeast display from a state-of-the-art naïve library, was reported to bind HSA with a *K*_d_ of 430 nM (*23*), which is in the same range as those of our de novo designs.

To confirm the binding, we also carried out biolayer interferometry (BLI) with immobilized HSA, obtaining *K*_d_ values compatible with those measured in solution (Fig. 3, D and E). The trypsin-targeting DesAb-Tryp used as a negative control gave no binding signal for HSA in this assay (Fig. 3D), while the yeast display–derived anti-HSA nanobody Nb.B201, used as a positive control, yielded a *K*_d_ compatible with that reported in the literature (fig. S4B) (*23*). DesAb-Tryp has the same sequence as DesAb-HSA-P1 and DesAb-HSA-P2, except for the designed CDR motif grafted in the CDR3 loop (table S1), and therefore represents a particularly suitable negative control to confirm that the observed binding is coming from the grafted designed motif. Besides, DesAb-Tryp was able to bind its intended target trypsin, while DesAb-HSA-P1 and DesAb-HSA-P2 showed no binding signal and were likely digested by the protease during the binding assay (fig. S5).

The crystal structure of DesAb-HSA-P1 in the unbound form further confirms the correct folding of the VH domain. This structure also reveals the dynamic nature of the CDR3 loop, which harbors the designed motif, as the electron density is missing for most of this region (fig. S3). A highly dynamic CDR3 loop was expected for this scaffold. For example, two of the four identical chains comprising the asymmetric unit of the structure of the original single-domain scaffold (PDB ID 3B9V) also have unassigned coordinates in their CDR3, even if the loop here is eight residues shorter than that of DesAb-HSA-P1. The highly dynamic nature of this loop likely stems from the lack of strong CDR3-framework contacts, which is why folding and stability of this scaffold have been shown to be insensitive to mutations in its CDR3 loop by several studies (*21*, *24*–*27*). We selected this scaffold precisely because it can harbor virtually any sequence in its CDR3 without marked consequences on its stability. However, the dynamic nature of the loop harboring the designed motifs likely also explains why we were unable to obtain a crystal structure of DesAb-HSA-P1 bound to HSA. We speculate that this dynamic loop, even when bound to the antigen, retains enough hinge flexibility to embody the resulting complex with a degree of dynamics unsuitable for structural determination.

In the absence of an atomic-level structural characterization of the designed interaction, we resorted to epitope binning through competition experiments. BLI competition experiments show that DesAb-HSA-P1 and DesAb-HSA-D3 compete with each other for binding to HSA, as the binding of one is hindered by the presence of the other antigen-bound DesAb (Fig. 3F). Conversely, DesAb-HSA-P2 does not compete with the other two, as its binding is not affected by the presence or absence of other antigen-bound DesAbs (Fig. 3F). This competition behavior is fully compatible with the rational design, as DesAb-HSA-D3 and DesAb-HSA-P1 were designed to target partly overlapping epitopes, while DesAb-HSA-P2 targets a different epitope on the opposite side of the antigen (Fig. 2D).

**Fig. 4.**
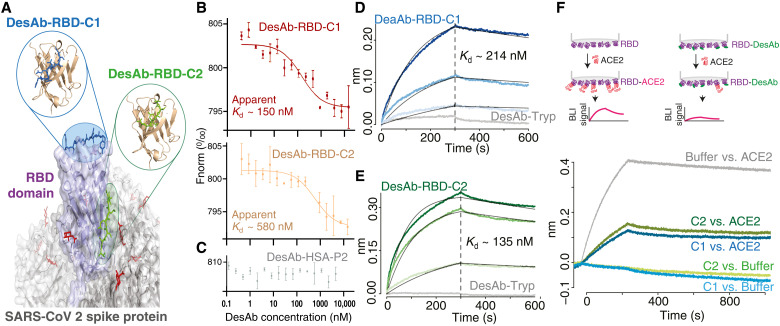
The anti-RBD DesAbs bind their target and compete with human ACE2. (**A**) The spike protein is shown in gray, and the RBD domain is shown in purple, with glycans in red. The designed CDRs are in blue and green for DesAb-RBD-C1 and DesAb-RBD-C2, respectively. (**B** and **C**) MST of Alexa Fluor 647–labeled trimeric spike protein (8 nM) in the presence of different concentrations of DesAb-RBD-C1 (red), DesAb-RBD-C2 (yellow), and DesAb-HSA-P2 (C; gray). Apparent *K*_d_ values are obtained by fitting a 1:1 binding model (see text). (**D** and **E**) BLI association and dissociation sensorgrams obtained with APS sensors loaded with RBD and blocked with HSA. Gray traces are obtained with 4 μM of DesAb-Tryp used as a negative control. (D) DesAb-RBD-C1 (4, 2, and 1 μM) (from darker to lighter blue, *K*_d_ = 214 ± 4 nM). (E) DesAb-RBD-C2 (4, 2.5, and 1 μM) (from darker to lighter green, *K*_d_ = 135 ± 2 nM). Data were fitted globally. (**F**) Binding competition experiment with sensors loaded like in (D) and (E) and dipped in wells with 5 μM DesAb-RBD-C1 (blue), DesAb-RBD-C2 (green), or buffer (gray), then in wells containing ACE2 or buffer controls (see legend), and finally back to buffer. The plot reports the last two steps, showing that the binding of ACE2 is substantially reduced by the presence of either DesAb-RBD-C1 or DesAb-RBD-C2 bound to the RBD.

Like the HSA-targeting DesAbs, the two designs made to target the RBD of the spike protein showed a binding affinity in the nanomolar range. We first tested the binding in solution to the full trimeric spike protein using MST (Fig. 4B; see Materials and Methods). Both RBD-targeting DesAbs showed binding to the spike protein, while the HSA-targeting DesAb-HSA-P2 used as a negative control gave no signal in the assay (Fig. 4C), confirming that the observed binding comes from the designed CDR3 motif. Fitting the binding curves with a 1:1 binding model reveals apparent *K*_d_ values of 150 and 580 nM for DesAb-RBD-C1 and DesAb-RBD-C2, respectively. As the spike protein is a trimer, a 3:1 binding model could have been, in principle, more suitable. However, while three distinct drops may be discernible in the binding curve of DesAb-RBD-C1, these are largely absent from that of DesAb-RBD-C2, and in both cases, the error bars are too large for a reliable 3:1 fit. To confirm the binding, we carried out a BLI assay with immobilized natively glycosylated RBD, which yielded *K*_d_ values of 210 and 130 nM for DesAb-RBD-C1 and DesAb-RBD-C2, respectively (Fig. 4, D and E). Conversely, these two anti-RBD antibodies showed no binding signal for immobilized HSA used as a negative control and as a blocker in the assay (fig. S4C; see Materials and Methods). We note that the lower apparent affinity of DesAb-RBD-C2 for the full spike, together with the absence of a three-step transition in its MST binding curve, is compatible with DesAb-RBD-C2 having a more sideway epitope (Fig. 4A), which may be poorly accessible in the down RBD conformation of the full spike (*19*). Last, both anti-RBD DesAbs were able to compete with the binding of the human ACE2 receptor to the viral RBD, which suggests that affinity-matured versions of these DesAbs may have neutralizing potential (Fig. 4F).

### Applicability of the design strategy

**Fig. 5.**
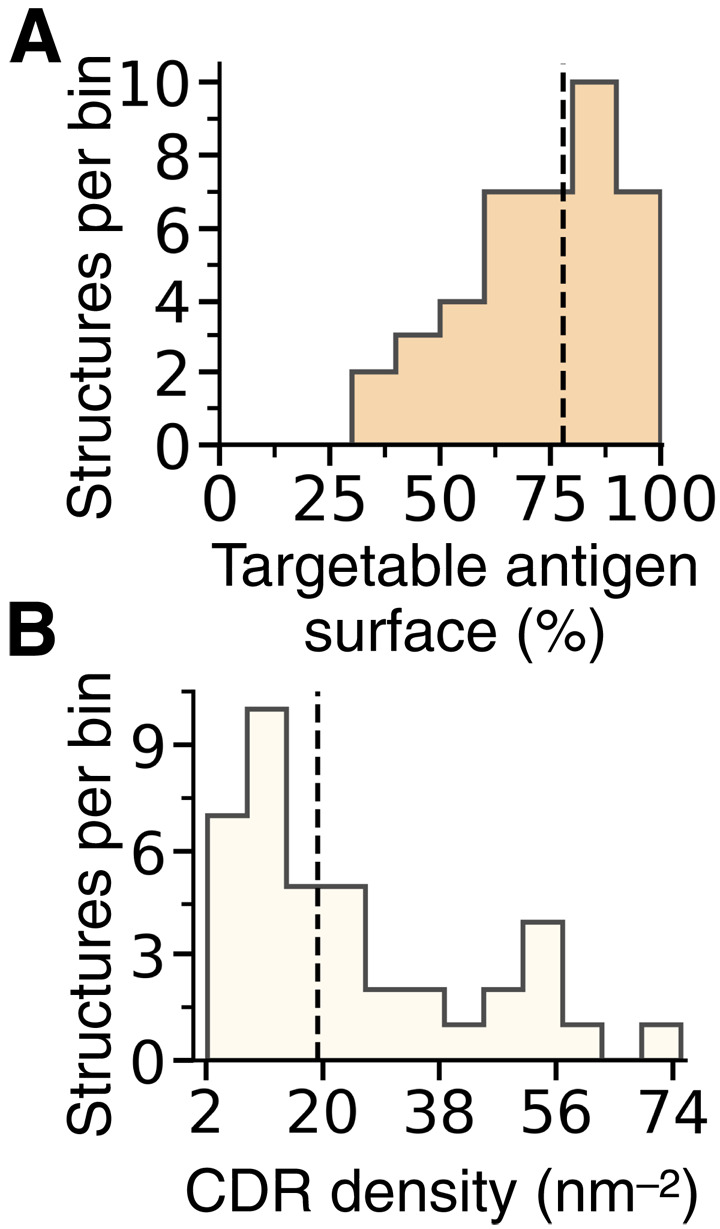
Applicability of the CDR design procedure. (**A**) Histogram of the distribution of the percent of targetable surface area for each antigen (experimental structures from CASP14). Targetable surface is defined as the SASA of the antigen made inaccessible by at least one designed CDR-like fragment. The dashed line is the median at 78%. (**B**) Histogram of the distribution of the CDR density for each antigen, expressed as the mean number of different designed CDRs per nm^2^ of antigen surface. The dashed line is the median at 19.2 CDRs per nm^2^.

Having established that our computational method can yield stable single-domain antibodies that bind their intended targets with *K*_d_ values down to the nanomolar range, we asked how readily and generally applicable the design strategy is. Given the fragment-based combinatorial nature of our method, we first asked what are the chances that suitable CDR-like fragments can be designed to target a given epitope, i.e., how typical it is for an epitope to have appropriate matching fragments in the AbAg database. To address this question, we run our design pipeline on the whole surface of all experimental target structures from the Critical Assessment of Techniques for Protein Structure Prediction competition (CASP14) (*28*). The target structures of the CASP assessments are selected ensuring that they represent a diverse sample of native folds, characterized by different sequences, secondary structures, and overall shape (*29*). Therefore, these structures also constitute a particularly suitable test set to explore the applicability of our design strategy. Having obtained all possible designed CDRs for each structure, we computed the solvent-accessible surface area (SASA) of the structure in the presence and absence of bound designed CDR fragments to reveal how much of the antigen surface is covered (see Materials and Methods). Our results reveal that most of the surface of each antigen is typically targetable with our strategy, with a median surface coverage of 78% (Fig. 5A). Furthermore, for each epitope, there are typically many candidate designed CDR loops to choose from, with a median density of 19 designed CDRs per nm^2^ of antigen surface (Fig. 5B). Together, these results reveal that, while some epitopes that cannot be targeted with our combinatorial strategy exist, most epitopes can be targeted by choosing between multiple different designed CDR candidates.

Having established that most of the epitopes can be targeted with our design strategy, the most apparent bottleneck of the pipeline is the need for a structure to be used as input. As structural determination can be challenging for some antigens, this aspect could limit the applicability of the method, in particular in the cases of emerging diseases or of poorly investigated areas, where novel antibodies are often most needed. Recent advances in structure prediction are changing this scenario, as it is now possible to readily obtain rather accurate models of most protein structures of interest (*30*, *31*). However, the accuracy of many methods of computational design, and in particular of those relying on energy functions that depend on interatomic distances, is known to rapidly deteriorate with lower-quality input models (*32*). Therefore, we next asked how applicable our method is on computationally modeled protein structures.

**Fig. 6.**
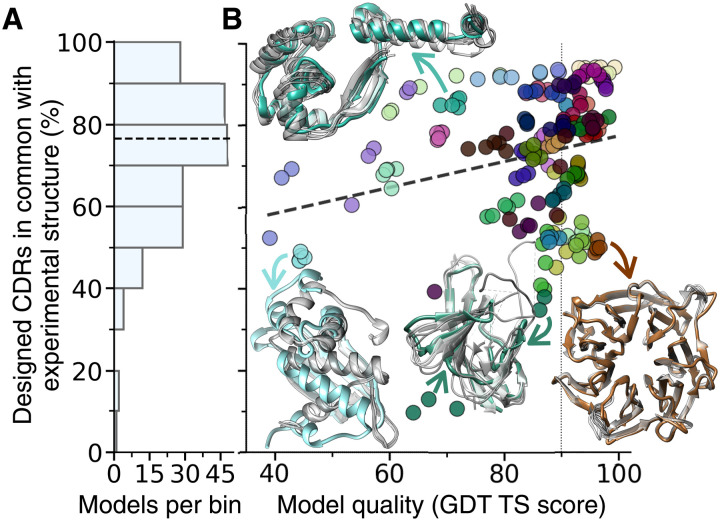
Performance on computationally predicted antigen models. (**A** and **B**) Computationally predicted models generated by AlphaFold2 within the CASP14 competition, as well as the corresponding experimentally determined structures, were used as input for the CDR design procedure. (A) Histogram of the distribution of the percent of designed CDRs obtained from each model that were identical to those obtained from the corresponding structure. The horizontal dashed line is the median of the distribution at 76.6%. (B) Scatter plot of the same CDR percent (*y* axis) as a function of the global distance test total score (GDT; *x* axis), which is an indicator of the model accuracy. GDT works with the percentage of ⍺-carbons that are found within certain cutoff distances of each other. A GDT of 100 means that the modeled and experimental structure have all ⍺-carbons within 1 Å of each other; a score above 90 (vertical dotted line) is typically considered a good solution of the folding prediction. The dashed trendline corresponds to a weak correlation (*R*^2^ = 0.06). Data points are colored according to the target experimental structure of each model (see table S3 and fig. S6H for a legend). Four example structures are drawn in the same color as their model data points, which are pointed by the arrows. Their models are overlaid to the structures and shown in gray.

To test the dependence of our design method on the quality of the input structural model, we ran our CDR design procedure on all CASP14 models generated with AlphaFold2, which was the best-performing algorithm assessed (*28*, *30*). By using all models deposited in CASP14 for each target structure, we also included in our analysis lower quality models that were not top ranking in CASP (see Materials and Methods). Our results reveal that most of the designed CDR-like fragments obtained by using each model as input are effectively identical to those obtained using the corresponding experimentally determined structure (Fig. 6A). More specifically, the median number of designed CDRs in common between each model and its corresponding experimental structure, expressed as a percent of the total number of designed CDRs obtained for each model, is 77%, and only 20 (10%) of the 200 models analyzed have less than 50% CDRs in common with their target structures (Fig. 6A, fig. S6, and table S3). These results suggest that if one were to use an AlphaFold2 model as input for our antibody design pipeline, typically about 75% of the generated CDRs would be identical to those that would be obtained from the corresponding crystal structure, and at least 50% would be identical in 90% of the cases. Besides, we only observe a very weak correlation (*R*^2^ = 0.06) between the percent of CDRs in common among model and structure and the quality of the model itself as quantified by the global distance test total score (GDT; Fig. 6B). This weak correlation indicates that the performance on modeled structures is not excessively determined by those very high-quality models (GDT ≥ 90) that are almost identical to their corresponding crystal structure. Together, these results imply that the CDR design procedure could be expected to yield similar results when running on computer-predicted models or on experimental structures, and that these results do not strongly depend on the quality of the model used as input, at least within the quality range we explored (GDT > 40).

## DISCUSSION

We have described a fragment-based strategy for the rational design of antibodies targeting structured epitopes. We use protein fragments of at least four residues and typically longer to assemble designed CDRs in a combinatorial way. The idea behind this choice is that these fragments should be large enough to contain nontrivial sequence determinants of structure and interactions (*6*, *21*, *33*).

Our experimental results demonstrate that the design pipeline that we presented can yield highly thermostable single-domain antibodies, which bind their intended targets with *K*_d_ values down to the nanomolar range (Table 1). This affinity range was confirmed with two independent experimental techniques, one relying on equilibrium thermodynamics in solution (MST) and one on binding kinetics with a surface-immobilized ligand (BLI). We explored slightly different design strategies, using single or multiple motifs to construct designed CDRs, and two grafting strategies (Fig. 2). In one, the designed motifs are grafted in the CDR3 of a stable scaffold, and in the other, they are structurally matched into two distinct loops of a compatible framework (DesAb-HSA-D3). We did not find substantial differences in the binding affinities of DesAbs obtained through different strategies.

We further verified, through various negative control experiments, that the DesAbs do not bind antigens that they are not intended for. Given that all DesAbs in this study, except for the two-loop design DesAb-HSA-D3, share the same framework sequence (table S1), these experiments make us confident that the observed interaction is coming from the designed binding motif grafted in the CDR loop. In a recent publication, we also show that dimeric conjugates of our anti-RBD DesAbs-RBD-C1 and DesAbs-RBD-C2 have 10- to 60-fold improved binding affinity toward the spike protein over their monomeric counterpart (*K*_d_, 8 to 15 nM), as one may expect from functional anti-RBD nanobodies (*34*). Furthermore, we observed a binding competition behavior fully compatible with the location of the target epitopes on the antigen surface (Figs. 3F and 4F).

Our failed attempts to obtain a structure of the bound complex, together with the structure of DesAb-HSA-P1 with missing electron density in the CDR3 region (fig. S3), suggest that these DesAbs differ from immune system–derived ones in their loop dynamics. This possibility is supported by recent results from molecular dynamic simulations, which compared the loop dynamic of DesAbs obtained with these grafting strategies with that of a nanobody obtained from llama immunization (*35*). Future work will be focused on addressing this limitation, to enable the design of rigid DesAbs amenable to structural characterization, which may even be applicable as crystallization chaperones like natural nanobodies (*36*), and also in assessing the immunogenicity of the designed antibodies.

We have been able to obtain DesAbs binding in the nanomolar range without the need of experimentally screening a large number of designs, but rather by preselecting in silico those designed CDRs that appeared most promising according to the metrics implemented, which include proxies for the predicted binding and side-chain complementarity, as well as predictions of solubility (see Materials and Methods) (*15*).

The fragment-based combinatorial approach presented here does not require the calculation of interaction energies and is also substantially faster than approaches based on the sampling of conformational and mutational space (*2*). Besides, this strategy is not highly sensitive to small variations in interatomic distances in the input model as, for example, force field calculations are, which helps explain why using models of varying quality for a given antigen results in similar CDR-like fragments (Fig. 5, C and D). An intrinsic limitation of this strategy, however, is that its applicability to epitopes of interest depends on the availability of suitable CDR-like fragments in the databases used. Nonetheless, the growing number of available protein structures in public databases makes the procedure generally applicable, as for most epitopes one obtains a number of candidate CDRs to choose from (Fig. 5 and fig. S1).

Our results, which are obtained with a computer code that can run on standard laptops, demonstrate that it is becoming increasingly possible to design de novo antibodies binding to preselected epitopes of interest. We have exploited recent advances in protein-folding predictions and ab initio structural modeling to show that our design pipeline yields similar results when running on experimental structures or on computer-generated models, even when these do not reach high accuracy. We envisage that, taken together, these advances in computational biotechnology will enable in the future to obtain lead antibodies in a matter of days from the release of a pathogen genome, or from the identification of a novel disease-relevant target.

## MATERIALS AND METHODS

### Data collection

All protein structures in the PDB (*37*) were downloaded from the rcsb.org website using a 90% sequence identity cutoff to reduce redundancy. Downloaded files were further cleaned by removing noncanonical amino acids and structures with no side-chain information. We refer to this dataset as the PDB90 database.

We further assembled a database of nonredundant CDRs, which we call CDR database. To create this dataset, the Structural Antibody Database (SAbDab) (*38*) was downloaded, and the structures of all heavy and light CDR types (CDR-H1,2,3 and CDR-L1,2,3) according to the Chothia definition were extracted from the antibody structures and filtered for redundancy. A database of antibody-antigen structures, filtered for peptide or protein antigens only, was also obtained directly from the SAbDab website and will be referred to as the Antibody-Antigen database. Last, a database of complete structures of antibody Fv regions, comprising both VH and variable light (VL) domains, as well as heavy chain–only antibodies (VHH) was retrieved from SAbDab and named Ab database.

### Generation of a database of antigen-CDR–like interactions

Each of the binding loop structures in the CDR database was used as query to look for structurally similar motifs in the PDB90 database. To achieve so, each template CDR loop of length *N* residues was fragmented using a sliding window approach with a range of [4, *N*] amino acids. Then, each of the generated fragments was matched against the whole PDB90 database using the MASTER program (*14*) (version 1.3.1) to find CDR-like structures. This structural search is based on the Kabsch algorithm (*39*), which uses RMSD of the carbon alpha positions. For that, an RMSD cutoff of 0.4 Å was used for fragments of length 4 and increased by 0.05 Å for each additional residue (the maximum cutoff value was set to 1.0 Å). In this way, we obtained a database of CDR-like fragments whose backbone is found in a conformation compatible to that observed in at least one known antibody CDR, but with no constraints on sequence similarity with known CDRs.

Next, we sought to establish whether these CDR-like fragments had an antigen-like partner region in their native environments (i.e., in the structures where they have been identified). Here, we define antigen-like region any part of a protein structure within the PDB90 database comprising one or more fragments of at least four consecutive residues that is in contact with a CDR-like fragment. Two different definitions of contacting residues were used: first, those residues in the structure whose calculated SASA increases upon removal of a CDR-like fragment; second, those fragments found within a distance of 10.5 Å between Cα atom pairs from a CDR-like fragment. Therefore, as a final product, two databases of antigen-like regions associated to the corresponding interacting CDR-like fragments were obtained, based on the two different residue-contact definitions. They will be individually referred to as the AbAg-SASA and AbAg-CACA databases and collectively as the AbAg database.

### Identification of CDR-like fragments interacting with a structured epitope

Given the structure of an epitope of interest as input, the two AbAg databases can be searched to identify antigen-like regions structurally similar to those within the input epitope. In this way, the CDR-like fragments interacting with the identified antigen-like regions have the potential to also interact with the structure of the epitope used as a query, as long as these regions have a reasonable sequence similarity. To perform this search, the structure of the epitope is fragmented into smaller regions to increase the probability of identifying matching antigen-like regions in the databases. Two fragmentation modes are used: The first one uses a sliding window approach to fragment contiguous peptides; window sizes are in the range [4, *N*], where *N* is the length of the input epitope, or the length of the epitope region under fragmentation in the case of input epitopes formed by multiple noncontiguous fragments. This fragmentation approach constitutes the “linear” mode. The other fragmentation mode takes each individual residue and calculates the closest *n* residues based on distances between the center of mass of their side chains (Fig. 1). This is done with various *n* values in the range [4, *N*]. This fragmentation approach constitutes a conformational mode, as it can readily generate regions comprising noncontiguous polypeptide segments that are close with each other in the input structure of the epitope. All the generated fragmentations of the input epitope are used as queries to interrogate the AbAg databases using the MASTER program doing full backbone-to-backbone comparisons using the same RMSD metrics as those used for the generation of the AbAg databases. To speed up the structural search, when using the linear fragmentation mode, the sequences of the generated epitope fragments are used as queries for a much faster blastp search (*40*) against the sequences of all antigen-like fragments within the AbAg databases (blast command: *blastp -query input_fragment_seauence.fasta -db AtAg_databases.fasta -qcov_hsp_perc 100.0 -matrixBLOSUM62 -task ‘blastp-short’ -word_size 2 -seg ‘no’ -evalue20000 -ungapped -comp_based_stats F -max_target_seqs 60000 -outfmt6 -out blast_hits.txt)*. This strategy is used to restrict the search space of the MASTER program within the AbAg databases to only those antigen-like regions with a sequence identity meeting a user-selected threshold. When using the conformational fragmentation mode, sequence identity is checked during the *AbAg* structural search whenever a match is found. In both modes, whenever a matching antigen-like region meets both sequence identity and structure similarity criteria, the corresponding interacting CDR-like fragments are retrieved. While the sequence identity threshold is specified by the user, the RMSD threshold (in Angstroms) is given by the function RMSDcutoff = 0.4 + n*0.033, where *n* represents the number of residues in the epitope fragment used as query. The retrieved CDR-like structures are then rotated to match the orientation of the input epitope by superimposing the matching antigen-like region together with its interacting CDR-like fragment(s) to the input epitope. As the matching region is typically smaller than the full input epitope, steric clashes may occur between the identified CDR-like fragments and the rest of the epitope or of the antigen, in which case the CDR-like fragments are discarded. Otherwise, these are labeled as CDR-like candidates (fig. S1).

### Optimization of the identified antigen-CDR–like interactions and raking of the hits

Each of the CDR-like candidates has a set of native interactions, which are defined as those interactions observed in the corresponding antigen-like region within the PDB90 database according to the SASA criterium of interaction described above. However, these interactions might not be fully conserved when the CDR-like candidate is paired with its corresponding epitope fragment, due to differences in amino acid sequence and side-chain orientation between the epitope fragment and the matching antigen-like region. If this is the case, the probability for the CDR-like candidate to interact with the epitope of interest might decrease. To address this issue, for each CDR-like candidate, we run an optimization procedure on those residues that have different interactions with the input epitope than the corresponding native ones. For each of these CDR-like residues, the optimization starts by defining a local interacting structural motif. This motif comprises all epitope residues that are found interacting with the CDR-like residue under scrutiny according to the SASA criterium of interaction described above. Next, this local structural motif, which includes also the backbone atoms of the CDR-like residue itself, is used as a query to look for similar regions in the PDB90 database (RMSDcutoff *=* 0.6 + n*0.025, where *n* is the number of residues). The aim is to find a matching region where the hit amino acid corresponding to the CDR-like residue under optimization has a backbone orientation very similar to the query, and therefore structurally compatible with the CDR-like candidate. Then, if those matching residues corresponding to the epitope residues have a sequence identity with the epitope higher than the current value, the side chain of the CDR-like residue is replaced with that of the new hit, always avoiding hits that cause steric clashes or proline and cysteine residues that may respectively alter the CDR backbone conformation or later cause covalent dimerization of designed antibody candidates. This procedure is applied to all CDR-like candidates that need it, to maximize the number of native interactions. As multiple residue positions within each CDR-candidate may be optimized, and as each of them may have multiple optimization options, all possible combinations are generated. For example, a candidate with three optimization options at position 1 and two options at position 4 will yield a total of 12 CDR candidates.

All candidates are ranked according to their solubility, as computed by the CamSol method (*15*). Furthermore, we also compute for each CDR-like candidate the number of native interactions, the number of shared interactions, and the number of interactions that are not shared, before and after optimization. Shared interactions are defined as interactions present in the original CDR-like/antigen pair found in the PDB90 database (native interactions) and that are also preserved in the optimized CDR-like bound to the epitope of interest. On the basis of these metrics, candidates with high number of shared interactions, low number of nonshared ones, and better solubility scores are regarded as the best ones. These scores and resulting rankings can be used to shorten the list of candidates and aid the selection of the most promising binding CDRs.

### Fragment assembly and CDR grafting

After optimization, the shortlisted CDR-like candidates are grafted in either full-length CDRs or directly full Fv antibody regions. At this stage, CDR-like candidates can also be combined with each other to obtain longer CDR candidates (Fig. 1). To do so, the first candidate of the shortlist is matched against the Ab or CDR database using MASTER, and the best match with no steric clashes between the epitope and the selected full CDR or complete Fv region is saved. Then, to combine together multiple CDR-like fragments in the same design, the same fragment is paired with all other fragments in the shortlist, and the pairs are matched against the Ab or CDR databases to see if both fragments could fit together different parts of the same CDR loop, or different CDR loops of the same Fv region. If any of the pairs of candidates is successfully matched, the result is taken to build triplets, and the matching process is repeated until no further match can be identified. After that, the process is repeated with the second candidate in the list, avoiding the already tested combinations. The iteration continues until all candidates and combinations are tested. Structural matching is done using Cα atom comparisons with RMSDcutoff *=* 0.4 + n*0.05, where *n* represents the total of residues in the query. This opens the opportunity of generating CDR loops comprising multiple CDR-like fragments, as well as antibodies with multiple candidates in different CDRs (fig. S1).

This joining and grafting procedure may introduce new interactions between the CDRs in which the candidates were grafted and the epitope, and possibly also between the epitope and other parts of the Fv region. If that is the case, each new set of interacting residues on the antibody side is subjected to the optimization procedure described above to increase the chances of successful binding. Last, the structure of the grafted candidates (either in CDRs or full Fv region) is produced as a final output.

### Generation of antibodies targeting HSA, SARS-CoV-2 spike protein, and trypsin catalytic site

The described algorithm was applied to the entire surface of HSA (PDB ID 1AO6, chain B), to the antigen binding region of the RBD of the SARS-CoV-2 spike protein (PDB ID 6VSB), and to a small region comprising the catalytic site of the trypsin protease (PDB ID 1S0Q). Both linear and conformational epitope fragmentation modes were used, with 70 and 60% sequence identity thresholds used during the CDR-like candidate search, respectively. The search was constrained to fragments of length 4 to 13 amino acids. Both AbAg databases were used. The list of CDR candidates was shortened by selecting those whose number of shared interactions was greater than the number of nonshared interactions and corresponded to at least two-thirds of the number of native interactions. For HSA, all shortlisted CDRs were then matched to full nanobody structures to find an amenable scaffold, and the top hit (based on the metrics describing the interactions, the solubility scores, and the quality of the grafting) was selected for experimental validation (DesAb-HSA-D3, consisting of two CDR fragments matched to the CDR1 and CDR3 of the VHH scaffold PDB 4DKA). In addition, the two top hits from the shortlisted CDR candidates (DesAb-HSA-P1 and DesAb-HSA-P2) were taken to be grafted directly into the CDR3 of a stable VHH scaffold (*21*). The latter strategy was also used for the spike RBD designs (DesAb-RBD-C1 and DesAb-RBD-C2) and trypsin active-site design (DesAb-Tryp).

### Analysis of the AlphaFold2 models and corresponding experimental structures within the CASP14 competition

Experimentally determined structures (targets) were downloaded from the CASP14 website, from https://predictioncenter.org/download_area/CASP14/targets/ (files therein were downloaded in January 2021 casp14.targets.T-dom.public_11.29.2020.tar.gz). AlphaFold2 models were downloaded from the same website using the Table Browser feature and by selecting “427 AlphaFold2” at https://predictioncenter.org/casp14/results.cgi?view=tb-sel and “all models” and “all targets.” Selecting “all models” instead of the default “model 1” is important as it enables to assess multiple models for each experimental target, including those that were not top ranking and hence have lower quality. This table also contained all the model quality metrics as calculated by the authors of the CASP14 competition, such as the RMSD and the GDT (table S3) (*29*, *41*).

Given that the experimental structures of some of the targets have not yet been released publicly, at the time of analysis, coordinates were available for 31 different targets of 63 expected from the table. As a consequence, we restricted our analysis to those 200 AlphaFold2 models that mapped on a target with available coordinates. These corresponded to five models per target, with the exception of three targets (T1024, T1030, and T1038) that had a total of 15 models each, as for these targets two domains had been independently modeled (five models per domain) and five additional complete models with both domains modeled together were generated, and of one target (T1050) that had a total of 20 models, as three domains had been independently modeled for it (again five models per domain plus five complete models). Furthermore, in a number of cases, there were amino acid residues present in the AlphaFold2 models, but not in the corresponding experimental structure (e.g., regions of missing electron density), or vice versa (residues not present in the model but present in the experimental structures). In these cases, we removed the extra residues before running the design calculations so that these ran on models and corresponding structures containing exactly the same residues (table S3, column “Processed”). This was a necessary precaution as the presence or absence of stretches of residues can generate different designed CDRs when running the design calculations. All PDB files from structures and models were cleaned using the PDBcleaner tool available on our web server (https://www-cohsoftware.ch.cam.ac.uk/) to remove HETATM and to grow any missing atom. It is worth noting that the authors of the CASP competition select their targets, also ensuring that they represent a diverse sample of native folds characterized by different secondary structure contents and overall shape, thus making these structures a particularly suitable test set to explore the generality of our antibody design strategy.

To obtain the results presented in the main text (Fig. 5), we ran our algorithm on the selected models and their corresponding experimental structures using a 70% sequence identity cutoff, and the requested minimum length of the CDR-like fragments was set to four residues. We then calculated the SASA for the entire input structure as well as for the structure in complex with all the identified CDR-like candidates. Subtraction of these values indicates the “surface coverage” per input structure (table S3).

In addition to the results presented in the main text, it is worth noting that the observation that data points corresponding to different models of the same structure tend to cluster together in Fig. 5B suggests that the nature of the antigen may play a bigger role in determining the robustness of the design procedure than the quality of the model itself. For example, the five lowest-ranking models, three of which are outlier in the distribution with less than 20% CDRs in common with their structure, are all for the same target (T1064 in table S3, PDB ID 7jtl, Fig. 5, A and B). This is a viral protein with a long disordered loop on one side and several missing residues, which are the main culprits for the large number of CDRs that are different among models and target. Furthermore, it is worth noting that (fig. S6): (i) the overall number of designed CDRs is typically very similar between models and target (Pearson’s *R* = 0.96), (ii) the fraction of designed CDRs that are obtained for the experimental structure and not for its models is typically small (median, 17%), and (iii) the total number of designed CDRs for a model appears to correlate with the overall fraction of CDRs that would also be obtained from the experimental structure (*R* = 0.51).

### Protein production and characterization

Genes encoding the anti-HSA single-domain antibody candidates (plus a C-terminal 7X His-Tag) were synthesized and cloned into an isopropyl-β-d-thiogalactopyranoside (IPTG)–inducible vector (by Atum in vector PD444) including a leading OmpA sequence to enable translocation to the periplasm and ultimately facilitate intradomain disulfide bond formation and the secretion of the product to the media. The anti-spike RBD and antitrypsin designs were introduced via restriction-free cloning into the CDR3 of the DesAb-HSA-P2 plasmid. For all DesAbs, versions with a free C-terminal cysteine residue were created using site-directed mutagenesis. This cysteine was inserted as part of an Asp-Cys-Glu motif right before the start of the C-terminal HisTag. For the anti-spike RBD designs, versions with a π-clamp sequence (FCPF) (*42*) followed by a TEV cleavage site right before the C-terminal 7× His-Tag were created by restriction-free cloning.

Plasmids were transformed into *E. coli* Shuffle LysY strain to further facilitate the formation of the disulfide bond of the antibody. Cultures (0.5 liter) of LB media were inoculated at initial 0.03 OD_600_ (optical density at 600 nm), grown at 37°C until reaching 0.8 OD_600_ nm, and then induced with 500 μM IPTG. Overnight expression was carried out at 30°C. Cellular pellet was discarded after centrifugation, and the supernatant was filtered using a 0.45-μm filter to remove remaining cell debris. Supernatant was passed twice through a gravitational flow column packed with Ni Sepharose Excel IMAC resin (Cytiva, 17-3712-01) previously equilibrated in phosphate-buffered saline (PBS) (pH 7.4). Then, the column was washed with PBS (pH 7.4), with a gradient of imidazole in PBS (10 and 30 mM). The protein was then eluted at 200 mM imidazole. Fractions were analyzed using SDS–polyacrylamide gel electrophoresis (SDS-PAGE), and those with the most protein and highest purity were dialyzed against PBS to remove the imidazole. Purified proteins were diluted to 20 μM, aliquoted, flash-frozen in liquid nitrogen, and stored at −80°C. The positive control nanobody Nb.B201 was expressed in the same way but using temperature and timing described in the original work (*23*), as well as the expression plasmid deposited in Addgene (pET26b_Nb.b201 Plasmid #131404).

### Antigens

HSA was purchased from Sigma-Aldrich (A3782) as lyophilized powder, resuspended in PBS, and further purified via gel filtration using a Superdex 200 size-exclusion chromatography column before use in binding assays. Pancreatic bovine trypsin was purchased from Sigma-Aldrich (T1426). Recombinantly produced [from human embryonic kidney (HEK) 293 cells] SARS-CoV-2 Spike Glycoprotein (S1) His-Tagged RBD was purchased from The Native Antigen Company (REC31849) and supplied to high purity in dry ice. SDS-PAGE analysis showed purity of >95% and a molecular weight consistent with the fully glycosylated RBD (data from The Native Antigen Company). Human ACE2 (18-615) recombinant protein, used in the competition assay in Fig. 4F, was also purchased from The Native Antigen Company, where it was expressed in HEK293 cells with Sheep Fc-Tag (REC31876). Trimeric His-Tagged SARS-CoV-2 Spike Glycoprotein was purchased from The Native Antigen Company (REC31871-100). Protein and antibody concentrations were determined by absorbance measurements at 280 nm using theoretical extinction coefficients calculated with Expasy ProtParam web server.

### Protein-thermal shift stability measurements

The melting temperature of the DesAbs was measured with a protein-thermal shift assay on a Bio-Rad CFX96 Touch quantitative polymerase chain reaction (PCR) machine using the ROX filter in white PCR plates. Samples were heated at 0.2°C/min from 25° to 95°C and consisted in purified antibody in PBS at a final concentration of 8 μM and of Sypro-orange dye in Protein Thermal Shift Buffer (Thermo Fisher Scientific, 4461146) accounting for 25% of the final volume at a final concentration of 2× the recommended dilution. Sample volumes of 50 μl per well were used. The signal from the dye in the absence of proteins was subtracted from the sample signal before analysis. The melting temperature (*T*_m_) was determined as the point of steepest derivative, and the values reported in fig. S2 are average and SDs over four replicates. In Table 1, we chose to round the *T*_m_ values to the closest 0.5°C because, while SDs across wells in the same plate are typically very small, interexperiment variations tend to be slightly larger.

### Circular dichroism

Far-ultraviolet (UV) CD spectra of the DesAbs were recorded using a Chirascan Applied Photophysics spectropolarimeter equipped with a Peltier holder, using a 0.1-cm path length quartz cuvette. Samples contained 6 μM protein in PBS. The far-UV CD spectra of all DesAbs were recorded from 200 to 250 nm at 25°C, and the spectrum of the buffer was systematically subtracted from the spectra of all DesAbs to yield the plots in fig. S2.

### Maleimide labeling

To obtain conjugates of the design antibodies to Alexa Fluor 647 dye, the C-terminal cysteine variants of the design antibodies were incubated with 1 mM dithiothreitol (DTT) for 10 min to reduce inter-DesAbs disulfide bond yielding covalent C terminus–C terminus dimers that may have formed during storage. DTT was then removed using Zeba desalting columns (Thermo Fisher Scientific, 89882), and samples were concentrated to 100 μM before incubation with Alexa Fluor 647–maleimide reagent (Thermo Fisher Scientific, A20347) for 1 hour at room temperature. Free dye was removed using PD-10 desalting columns (Cytiva, 17-0851-01), and the labeling efficiency was assessed by absorbance measurements. Trimeric SARS-CoV-2 spike protein was fluorescently labeled by incubating a 2.8 μM protein solution with 50 molar equivalents of Alexa Fluor 647–*N*-hydroxysuccinimide ester reagent (Thermo Fisher Scientific, A20006) in the dark during 2 hours at room temperature. Excess dye was removed by desalting three times with Zeba columns (Thermo Fisher Scientific, 89882), and labeling efficiency was determined by absorbance (estimated to be 13:1 dye to protein labeling ratio).

### MST binding affinity measurements

For the anti-HSA designs, starting from 30 μM HSA (150 μM for the KK5 control DesAb in fig. S4), 16 samples of 1:1 serial dilutions were incubated with 70 nM Alexa Fluor 647–labeled antibody for 1 hour at room temperature. Samples were prepared in 170 mM NaCl, 50 mM tris-HCl, 10 mM MgCl_2_ (pH 7.4) with 0.05% Tween 20. After incubation, samples were run in triplicate in the Monolith NT.115 System (NanoTemper Technologies) using 20% light-emitting diode (LED) excitation power and 60% MST power, at 25°C. For the anti-RBD designs: DesAb-RBD-C1 and DesAb-RBD-C2 (variants with the C-terminal π-clamp and TEV cleavage site) at 14.4 μM and the anti-HSA control DesAb-HSA-P2 at 4 μM were used as starting concentration for preparing 16 1:1 serial dilutions in PBS (pH 7.4) with 0.05% Tween 20. They were incubated with a final concentration of 8 nM Alexa Fluor 647–labeled trimeric SARS-CoV-2 Spike protein at room temperature for 1 hour. After incubation, samples were run in triplicate in the Monolith NT.115 System (NanoTemper Technologies) using 15% LED excitation power and 80% MST power, at 25°C. All data were analyzed and fitted using the Monolith System software assuming a 1:1 binding interaction.

### BLI binding affinity measurements

BLI measurements were performed using an Octet-BLI K2 system (ForteBio). All assays were carried out in a black 96-well plate, 200 μl per well, and all sensors were subjected to prehydration in the assay buffer for at least 15 min before usage. The assay plate was kept at 25°C throughout the entire experiment. For consistency with the MST measurements, anti-HSA design binding assays were carried out in a buffer containing 170 mM NaCl, 50 mM tris-HCl, and 10 mM MgCl_2_ (pH 7.4). First, two aminopropylsilane (APS) sensors (sample and reference) were preincubated in buffer for 15 min. Assay program consisted of a 150-s baseline in buffer; 300-s loading using 4 μM HSA; 300-s wash in buffer; 90-s baseline in buffer; 300-s association in 1 μM, 500 nM, and 250 nM anti-HSA DesAbs for the sample sensor and buffer for the reference sensor; and 300-s dissociation in buffer (Fig. 2D). As a control for nonspecific binding to the sensors, the same experiment was carried out with the DesAb-Tryp instead of the anti-HSA DesAbs (Fig. 2D). The positive control Nb.B201 experiment was carried out in the same way but using 800 and 400 nM as analyte concentrations (fig. S4B). Binding competition experiment of the anti-HSA designs was carried out in a similar way, in a buffer consisting of one-third of PBS pH 7.4 and two-thirds of the aforementioned 50 mM tris-HCl, 170 mM NaCl, and 10 mM MgCl_2_ (pH 7.4). APS sensors were loaded with HSA for 600 s, baseline for 300 s, then dipped in wells containing 5 μM of a first DesAb X1 for 600 s, moved in buffer wells for 60 s, then into wells containing 5 μM of a second DesAb X2 for 300 s, and finally back to buffer wells for 600 s to monitor dissociation. DesAbs X1 and X2 refer to different combination of the anti-HSA DesAbs as in the legend of Fig. 2F. Because trypsin could not be loaded effectively on APS sensor, the trypsin binding assay was carried out with Ni–nitrilotriacetic acid sensors using the same buffer composition as above. Sensors were loaded with 7.5 μM His-tagged DesAb-Tryp or control DesAb (either DesAb-HSA-P1 or DesAb-HSA-P2 as in fig. S5) for 900 s. We found that loading these sensors to saturation was the only viable way to fully suppress the nonspecific binding of trypsin to the nickel sensors; hence, we systematically used control DesAbs for all trypsin concentrations tested. These controls are identical to DesAb-Tryp except for the designed CDR3 (Table 1). Following loading, a baseline was taken for 180 s and then association and dissociation steps as in fig. S5. Assays for DesAb-RBD designs were carried out in PBS with APS sensors, following the program: 120-s baseline in buffer, 90-s loading using 400 nM RBD, 300-s 4 μM HSA blocking, 120-s baseline, 300-s association, and 300-s dissociation.

Data with multiple DesAb concentrations were fitted globally with in-house python scripts, using *K*_on_ and *K*_off_ as global fitting parameters and *R*_max_ as a local parameter (i.e., each DesAb concentration was allowed its own value of *R*_max_ as these are probed by different BLI sensors). The *K*_d_ was then derived as the ratio of *K*_off_/*K*_on_. Because of the shape of its dissociation curve, the positive control Nb.B201 experiment was fitted with a model that does not assume full dissociation at infinite time (fig. S4B).

### Crystallization, data collection, data reduction, structure determination, refinement, and final model analysis

DesAb-HSA-P1 was concentrated before the setup of crystallization trials to a final concentration of 10 mg/ml. Crystals of DesAb-HSA-P1 were obtained with the vapor diffusion technique, in sitting drops, using equal volumes of the protein and 0.1 M sodium cacodylate (pH 6.5), 27% Polyethylene glycol 2000 monomethyl ethers (PEG-MMEs) as a precipitant solution. Diffraction data were collected on cryoprotected crystals (25% glycerol) at 100 K, at the I04 beamline of the Diamond Light Source using a 0.9795-Å wavelength. The collected dataset was processed with Dials (*43*) and Aimless (*44*) from the CCP4 suite (*45*). The structure was solved by molecular replacement with Phaser (*46*) using as a search model PDB ID 3B9V. The correct amino acids of the DesAb-HSA-P1 construct were built manually using COOT (*47*, *48*). The initial model was refined alternating cycles of automatic refinement using Phenix (version 1.17_3644) (*49*) and manual model building in COOT. Data collection and refinement statistics are reported in table S2. Analysis of molecular interfaces was performed using PISA (*50*).

## Notes

View/request a protocol for this paper from *Bio-protocol*.
