# Supplementary material for: Fragment-based computational design of antibodies targeting structured epitopes
Source: Sci Adv. 2022 Nov 11;8(45):eabp9540. doi: 10.1126/sciadv.abp9540 (PMC9651861; doi:10.1126/sciadv.abp9540)
Supplement: Supplementary file 1 — Figs. S1 to S6 Tables S1, S2 and S4 [file sciadv.abp9540_sm.pdf]

Science Advances
AAAS

Supplementary Materials for

Fragment-based computational design of antibodies targeting structured epitopes

Mauricio Aguilar Rangel *et al.*

Corresponding author: Pietro Sormanni, ps589@cam.ac.uk; Michele Vendruscolo, mv245@cam.ac.uk

Sci. Adv. **8**, eabp9540 (2022)
DOI: 10.1126/sciadv.abp9540

The PDF file includes:

Figs. S1 to S6
Tables S1, S2 and S4
Legend for table S3

Other Supplementary Material for this manuscript includes the following:

Table S3

DesAb scaffold<br>(PDB ID 6Z3X)	MEVQLEESGGGLVQPGGSLRLSCAASGFNIKDTYIGWVRQAPGKGEEWVASIYPTSGYTRYADSVK<br>GRFTISADTSKNTAYLQMNSLRAEDTAVYYCAAGS**_DesignedCDR3_(GS)**EEFDYWGQGTLVT<br>VSS<br>DHHHHHHH
DesAb-HSA-D3	MEVQLQESGGGLVQAGGSLRLSCAASG_**ELYALI**_SMGWFRQAPGKEREFVAAISRNGDNTYYTDS<br>VKGRFTISRDNAKNTVELQMNSLKPEDTAVYYCAAD_**KFASPDGS**_VTIMTNEYDYWGQGTQVTVS<br>DHHHHHHH

Table S1. **Amino acid sequence of the single-domain antibodies employed in this study.** In the sequence in the top row "_DesignedCDR3_" should be replaced with the designed CDR sequences reported in **Table 1** of the main text to obtain the sequence of each DesAb. In the case of DesAb-RBD-C1 and C2 only, the further motif GS (in round brackets) should be added. This is not part of the designed CDR motif, but it extends the CDR3 to better accommodate the extended motif of these two DesAbs. The PDB ID 6Z3X is from this study. The 7x-His tag was used for purification. All DesAbs have a OmpA signal peptide, which is cleaved upon translocation to the periplasm yielding the sequences in the table.

DesAb-HSA-P1	
**Data collection**	
Wavelenght (Å)	0.9795
Space group	P2(1)2(1)2(1)
Unit cell parameters	
a, b, c (Å)	40.72, 52.41, 99.18
α, β, γ (°)	90, 90, 90
Wavelength (Å)	0.9795
Resolution (Å)	46.34-1.74<br>(1.77-1.74)
Number of unique reflections	45160 (2432)
R_{merge}	0.108 (0.776)
R_{meas}	0.121 (0.873)
R_{pim}	0.053 (0.396)
<I/σ(I)>	8.7 (1.5)
$CC^{1/2}$	0.998 (0.915)
Completeness (%)	100 (99.4)
Multiplicity	4.7 (4.7)
**Refinement**	
Resolution (Å)	46.34-1.74
Number of reflections	42002
R_{work}/R_{free} (%)	19.25 / 21.96
r.m.s. deviations	
bond length (Å)	0.010
bond angles (°)	1.049
Ramachandran plot	
favored (%)	99
allowed (%)	1
outliers (%)	0

Table S2. Data collection and refinement statistics related to the structural determination of DesAb-HSA-P1. Values in parentheses refer to the highest resolution shell.

Table S3 (Included as separate tab-separated file). AlphaFold2 models and corresponding experimental structures from CAPS14 employed in this study. This file can be opened with most spreadsheet editors including Microsoft Excel. Table with refernce to all model pdb files associated with their corresponding experimentally determined structures (Target pdb files). The model-quality scores in the columns 'GDT_TS', 'GDT_HA', 'RMS_ALL', and 'RMSD' were downloaded from the CASP14 website as calculated by the CASP authors. The column 'TP' (True Positives) reports the number of designed CDRs that could be obtained by using as input either the model or the experimental structure, 'FP' (False Positives) the number of designed CDRs obtained from the model but not from the stucture, and 'FN' (False Negatives) the number of designed CDRs obtained from the structure but not from the model. A small number of files (column 'Processed' set to 'Y') was processed by us to remove some residues that were present in the model and not in the structure or vice versa (see columns 'ResCoverage.of.original.model'and 'ResCoverage.of.original.structure'). All strutures and models were downlaoded from the CASP14 website (see Methods). 'precision', which is equal to TP/(TP+FP), is the fraction of CDRs obtained from the model that can also be obtained from the structure.

Source structure	Sequence
5vnw	ChainD 101RSYSFR
5vnw	ChainD 96AVLETRS
5vnw	ChainD 55TNTYYA

Table S4. **Nanobody-derived anti-HSA CDR-like hits obtained by running our method on the surface of HSA.** These fragments, which we didn't use in any of our designs, are actually derived from an existing Nanobody-HSA complex included among the structures used to construct the Antibody-Antigen database (AbAg database). We note that other structures of HSA-bound antibody fragments were not parsed during the creation of the AbAg database, as these were not contained in the PDB90.

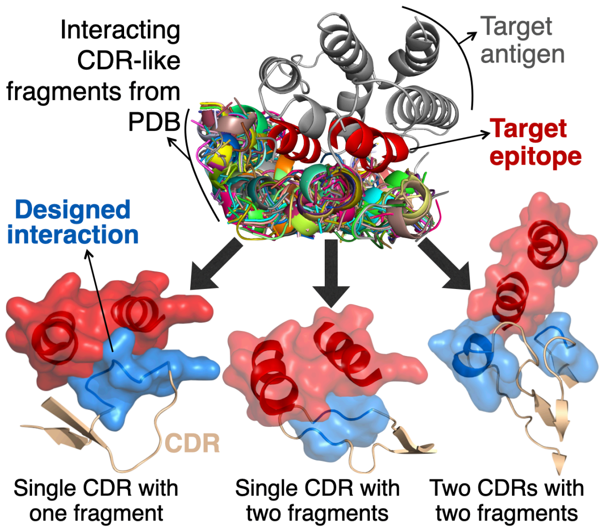

Figure S1. Examples of designed CDRs to target conformational epitopes. The target antigen is shown in grey at the top and the selected target epitope in red. Candidate interacting fragments (multiple colours) are selected as those fragments with a CDR-like backbone structure (e.g. backbone coordinates compatible with those of natural antibody CDRs) that are found in the Protein Data Bank (PDB) interacting with at least one fragment whose sequence and backbone structure match those of a corresponding fragment within the target epitope (see Methods). The lower panels, which are also reported in Fig. 2A of the main text, show three examples of how these CDR-like fragments can be combined in different ways to design interacting motifs (light blue), which are grafted onto structurally matched CDR loops (light brown) to generate lead antibodies predicted to bind to the target epitope.

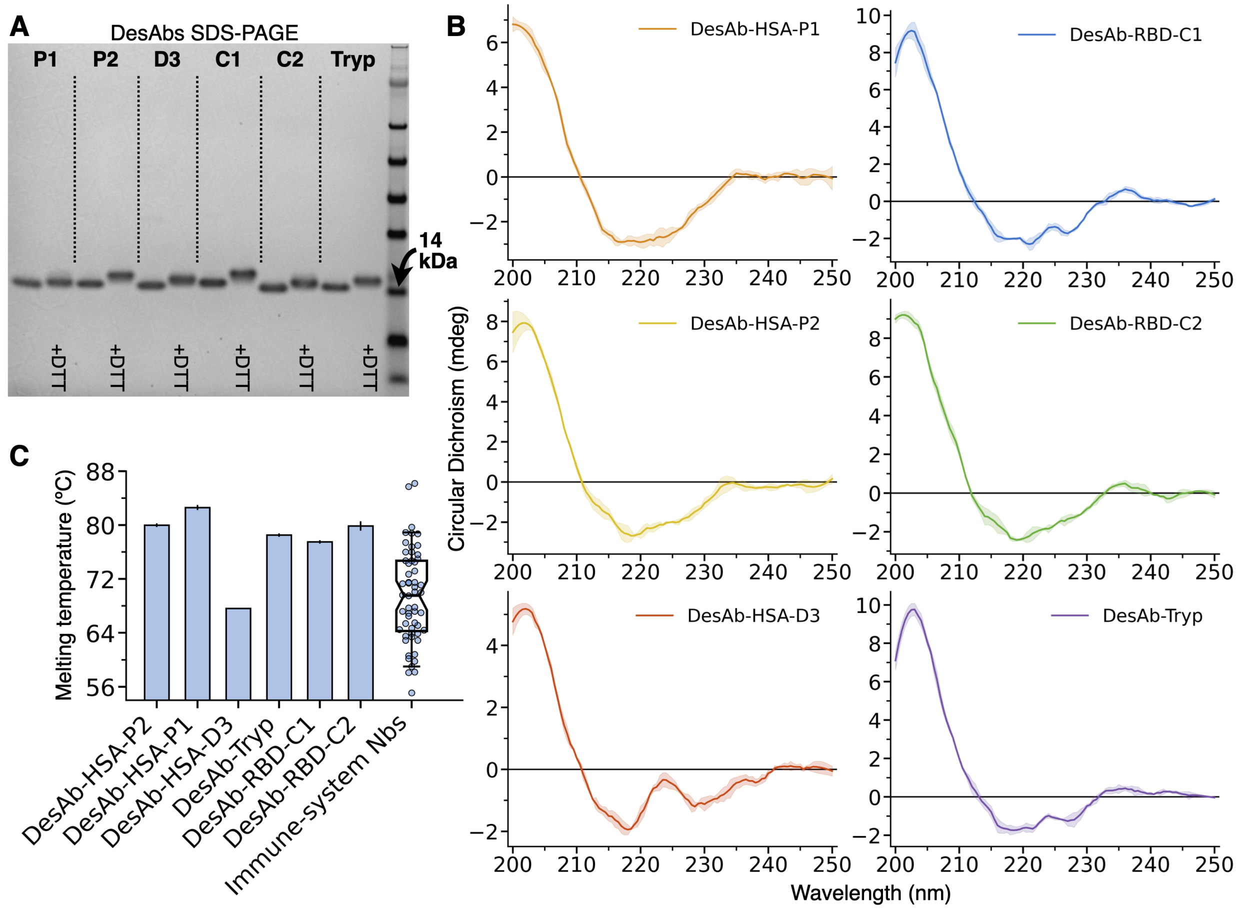

Figure S2. Biophysical characterisation of the designed single-domain antibodies. (**A**) SDS-PAGE of the six DesAbs designed in this study taken after purification (see legend), dashed vertical lines are guides for the eyes. The small difference in migration between samples without and with reducing agent (+DTT) indicates the correct formation of the intra-domain disulphide bond. (**B**) CD spectra of the six DesAbs (see legend) showing the expected minimum at 218 nm characteristic of β-domains. Profiles were smoothed using Savitsky-Golay filtering, and the shaded area is the standard deviation of three technical replicates. The spectrum of DesAb-HSA-D3 is substantially different from the others, as this design was done following a different strategy employing a different single-domain antibody scaffold for the grafting (see main text). (**C**) Bar plots with the melting temperatures of the six DesAbs measured with a protein thermal-shift assay (see Methods), compared with those of 68 nanobodies (swarm-plot to the right) isolated from camelids and reported in Ref. [20]. The box represents the first and third quartiles of the distribution of these 68 melting temperatures, whiskers represent the 1.5 interquartile range, and the bar at the centre the median.

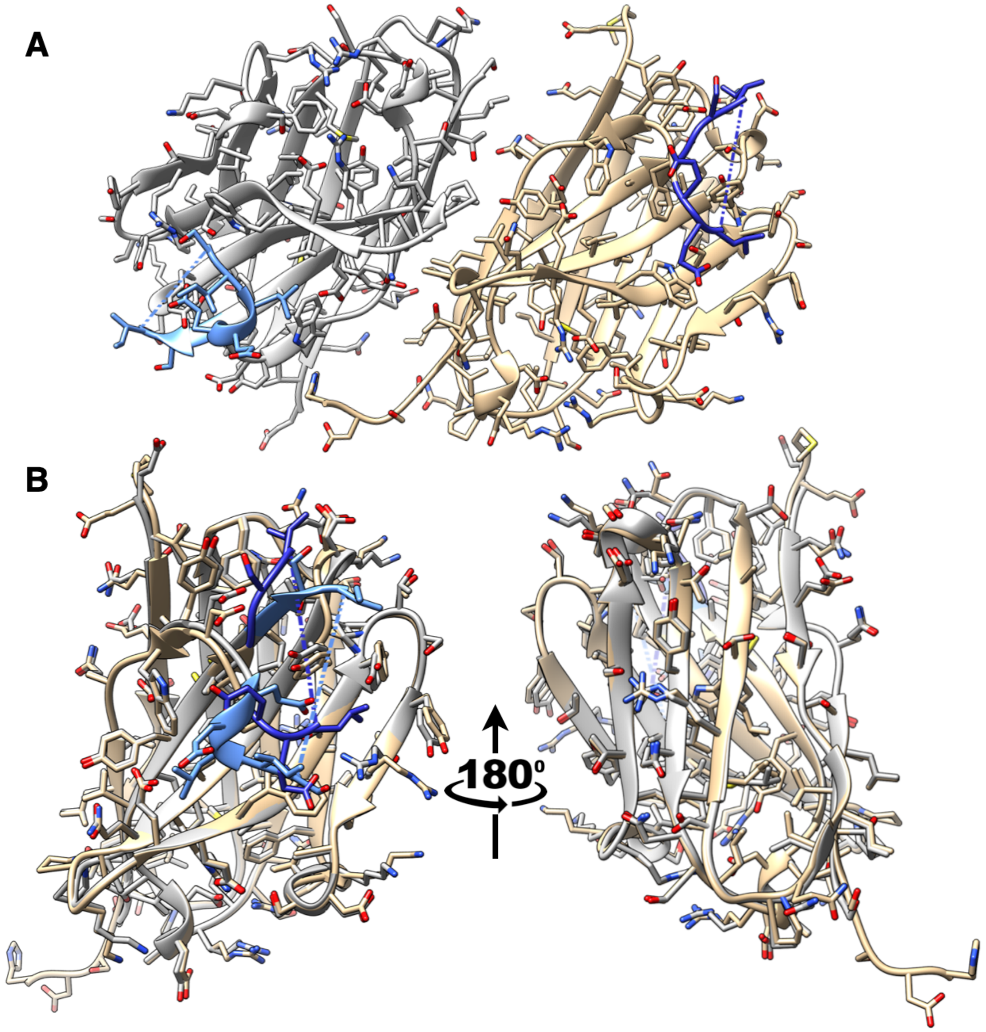

Figure S3. Crystal structure of DesAb-HSA-P1. (**A**) Asymmetric unit containing two single-domain antibodies, chain A is in grey and B in brown. The CDR3 loops that harbour the designed motifs are coloured in blue. Dashed lines denote a stretch of missing residues corresponding to the lower-case residues in the motif: `GSIqkslqtaeSILEE` (light blue in chain A) and `GSIqkslqtaesiLEE` (dark blue in chain B). (**B**) Structural superimposition of chain A and B from the asymmetric unit further reveals the dynamic nature of the CDR3 (blue), whose stems are found in substantially different conformations in the two single-domain antibodies. Coordinates have been deposited in the PDB with accession code 6Z3X.

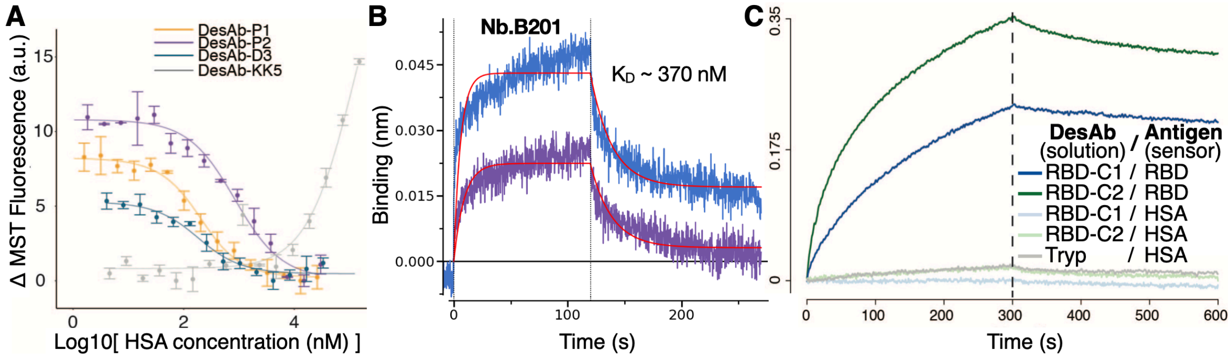

Figure S4. Control experiments for the binding of the DesAbs to their targets. (**A**) MST traces from **Fig. 3A-C** with the trace of an additional control single-domain antibody (KK5 in grey) targeting an unstructured epitope in an unrelated antigen (the human tau protein) and obtained as described in Ref. [7]. This single-domain antibody was fluorescently labelled with Alexa647 at an engineered solvent-exposed cysteine exactly like the other DesAbs (see Methods), and it is based on the same scaffold as DesAb-HSA-P1 and DesAb-HSA-P2 (VH-domain sequence identity respectively of 89% and 92%). For KK5 the signal deviates from flat at HSA concentrations >10 μM possibly because of molecular crowding and/or non-specific binding. Data for this control can be fitted with a $K_D \geq 110$ μM, which is only a lower bound as these data points do not reach a second plateau. (**B**) BLI experiment carried out exactly like those in **Fig. 3D** but using a positive control nanobody as analyte. The fitting model employed here does not assume full dissociation at infinite time and the KD obtained is in broad agreement with that reported in the literature (KD ~ 430 nM) [11]. (**C**) BLI experiment like in **Fig. 4D,E** using 4 μM of DesAbs in solution showing also binding traces obtained with different antigens on the sensors (see legend).

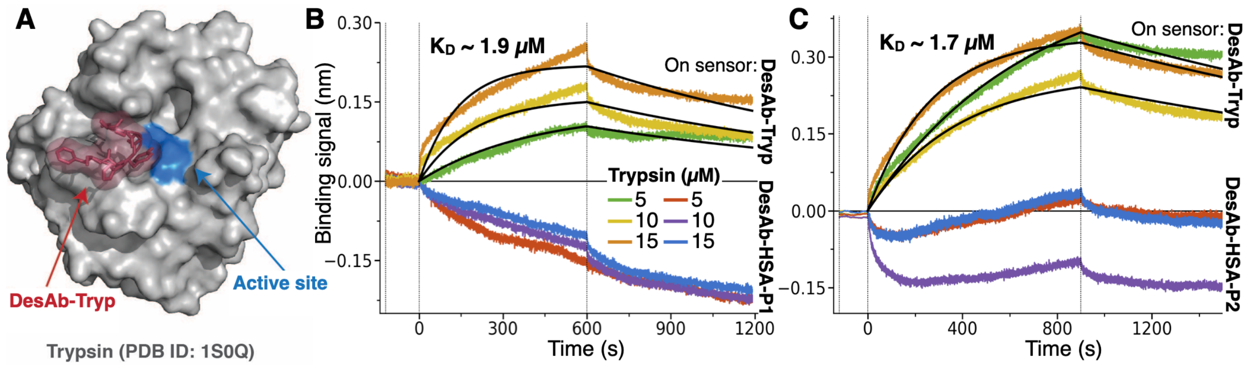

Figure S5. Binding of DesAb-Tryp to trypsin. (**A**) The structure of pancreatic bovine trypsin is shown in grey in surface representation (PDB ID 1S0Q), and the designed CDR fragment targeting an epitope within the active site (blue) is represented in dark red. This fragment is then grafted onto the CDR3 of DesAb-Tryp (**Table 1**). While running this design calculations, care was taken to exclude all known peptide inhibitors of trypsin or related proteases whose structure is available in the PDB. Indeed, because of their perfect complementarity with the active site of trypsin, such peptides figured as top-ranking, but testing them experimentally within a CDR loop would have defied the purpose of using our fragment-based combinatorial approach to design novel interactions. (**B**, **C**) BLI assays carried out with Ni-Nta sensors loaded with DesAb-Tryp (yellow, green and orange traces) or DesAb-HSA-P1 or DesAb-HSA-P2 (blue, red and purple traces, respectively in **B** or **C**). Panel **B** and **C** correspond to two independent experiments carried out in different days with a different choice of negative control DesAb. Shown are the end of the baseline phase, the association and dissociation phase separated by vertical dashed lines. Different Trypsin concentration are present in the association phase (see legend). Data from sensors loaded with DesAb-Tryp were fitted globally (see Methods), yielding a mean K_D for trypsin of 1.8 μM. The decreasing signal observed for the negative control DesAbs is consistent with the trypsin protease digesting these V_{HH}s, which are immobilised on the sensor surface via their C-terminal His-tag. DesAb-HSA-P1is likely digested more, as, unlike DesAb-HSA-P2, it has a lysine in its designed CDR3 (**Table 1**). DesAb-Tryp was not characterised further as we observed that, in solution, this was also digested by the trypsin protease.

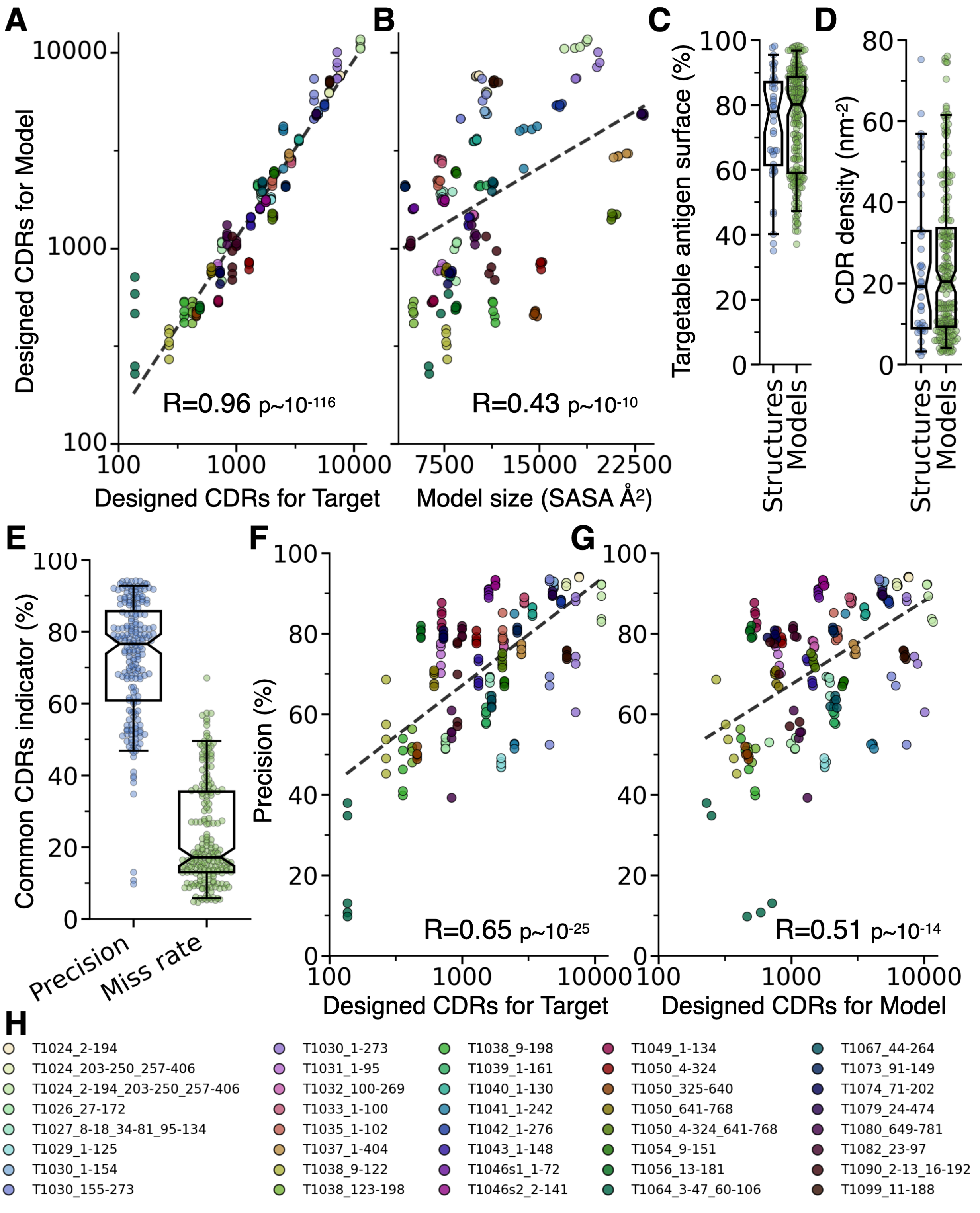

Figure S6. Additional analysis of CDR-design on computationally predicted antigen structures. (**A**) Scatter plot with the total number of designed CDRs that could be obtained when running the procedure using as input each structural model (y-axis) or its corresponding

experimentally determined target structure (x-axis). (**B**) Total number of designed CDRs for each model as a function of the model size, expressed as its solvent-accessible surface area (SASA, x-axis). (**C**) Swarm plots of the percent of the input antigen surface that can be targeted with at least one designed CDR, and of the CDR density (**D**), calculated as the average number of designed CDRs per nanometre squared of the antigen surface. (**C**, **D**) Both quantities are calculated for all experimental structures (blue, same distributions as in **Fig. 5C** and **D** respectively) and all models (green). (**E**) Swarm plots of the percent of designed CDRs from each model that were identical to those from the corresponding target structure (Precision, same distribution as in **Fig. 5A**) and of the percent of designed CDRs from the target structure that were not found when running on each of its models (Miss rate). Boxes represent the first and third quartiles of the distribution, whiskers represent the 1.5 interquartile range, and the horizontal bar at the centre the median. (**F, G**) Scatter plots of the precision as a function of the total number of designed CDRs for each target structure (**F**) and for each model (**G**). In all scatter plots R is the Pearson coefficient of correlation and p its corresponding p-value. (**H**) Legend of the colour of the markers in this figure and in **Fig. 5**, which are coloured according to the identity of the target structure of each model (see **Table S3**). Labels are the CASP14 target ids underscore the residue-range that was modelled in each model.
